# Correction: X-ray absorption near edge structure simulation of LiNi_0.5_Co_0.2_Mn_0.3_O_2_*via* first-principles calculation

**DOI:** 10.1039/d0ra90002h

**Published:** 2020-01-23

**Authors:** Toshiharu Ohnuma, Takeshi Kobayashi

**Affiliations:** Material Science Research Laboratory, Central Research Institute of Electric Power Industry (CRIEPI) 2-6-1 Nagasaka Yokosuka-shi Kanagawa-ken 240-0196 Japan ohnuma@criepi.denken.or.jp

## Abstract

Correction for ‘X-ray absorption near edge structure simulation of LiNi_0.5_Co_0.2_Mn_0.3_O_2_*via* first-principles calculation’ by Toshiharu Ohnuma *et al.*, *RSC Adv.*, 2019, **9**, 35655–35661.

The author regrets that the funding information was incorrectly shown in the Acknowledgements section of the original manuscript. The corrected funding acknowledgement is as shown below.

The synchrotron X-ray absorption near-edge structure measurements were performed with the approval of the Japan Synchrotron Radiation Research Institute (JASRI) Program Advisory Committee (Proposal No. 2014A5350, 2014B5350, 2015A5350, 2015B5350, 2016A1567, 2016A5350, 2016B5350, 2018A1596, 2018A5350, 2018B1593, 2018B5350, 2019A1818 and 2019A5350).

The Royal Society of Chemistry apologises for these errors and any consequent inconvenience to authors and readers.

## Supplementary Material

